# Where are the parasites in food webs?

**DOI:** 10.1186/1756-3305-5-239

**Published:** 2012-10-23

**Authors:** Michael VK Sukhdeo

**Affiliations:** 1 Department of Ecology, Evolution and Natural Resources, Center for Research on Animal Parasites, Rutgers University, New Brunswick, NJ, 08901, USA

**Keywords:** Food webs, Parasites, Energetics, Matrix models, Topology, Network theory

## Abstract

This review explores some of the reasons why food webs seem to contain relatively few parasite species when compared to the full diversity of free living species in the system. At present, there are few coherent food web theories to guide scientific studies on parasites, and this review posits that the methods, directions and questions in the field of food web ecology are not always congruent with parasitological inquiry. For example, topological analysis (the primary tool in food web studies) focuses on only one of six important steps in trematode life cycles, each of which requires a stable community dynamic to evolve. In addition, these transmission strategies may also utilize pathways within the food web that are not considered in traditional food web investigations. It is asserted that more effort must be focused on parasite-centric models, and a central theme is that many different approaches will be required. One promising approach is the old energetic perspective, which considers energy as the critical resource for all organisms, and the currency of all food web interactions. From the parasitological point of view, energy can be used to characterize the roles of parasites at all levels in the food web, from individuals to populations to community. The literature on parasite energetics in food webs is very sparse, but the evidence suggests that parasite species richness is low in food webs because parasites are limited by the quantity of energy available to their unique lifestyles.

## Introduction

Over the last decade, there has been an enormous increase in the number of publications dealing with parasites and food webs, and there is no longer the need to argue that parasites must be included in all models of ecosystem function. The credit for this transformative change goes to a 1997 paper by Marcogliese and Cone [1] who made a strong plea for including parasites in food webs, and they compiled a long list of convincing arguments for the utility of parasites in clarifying or explaining diverse food web phenomena. Their paper started a gold rush in the field! Nevertheless, despite all of the effort expended since then, we are no closer to an understanding of parasites in food webs than we were in 1997. Even though more papers on the subject are being written than ever before, few generalizations have emerged, and there still seems to be no satisfactory way to insert parasites into modern food web theory [2-6]. For example, key assumptions, such as size-based trophic cascades, disallow parasites in food web models because parasites are smaller than their food (hosts), and incorporating them into food web models creates looping errors [1,4,5]. Consequently, although parasites are generally excluded from food web analyses, they are frequently mapped *post hoc* onto food web diagrams [7,8]. Or, if included in food web matrices as distinct nodes, parasites are incorrectly treated as predators or trophospecies for analytical ease [7-10]. This difficulty in fitting parasites into the fundamental framework of food web biology is recognized as a major stumbling block [2,4,10-14]. However, while it may appear to be a huge obstacle to progress, this situation also presents us with a rare opportunity to question the critical assumptions that underlie our understanding of natural communities.

### The parasitological perspective on food webs

The areas of study that encompass food web biology and parasites are vast and complex, and this review cannot be fully comprehensive. However, although this review is limited to inferences on helminth (worm) parasites in food webs, the ideas are also relevant to many other organisms that are not traditionally included in food webs, including prions, viruses, bacteria, plankton, protozoa and fungi. This review is also primarily aimed at an audience of traditional parasitologists, and especially graduate students who might be interested in exploring the rapidly expanding fields of parasite ecology and evolution. Readers are not expected to be ecologically-proficient, nor are they expected to be very experienced in the assessment of mathematical models. This essay will paint with broad strokes, and the discussion will be restricted to verbal models rather than mathematical models (although it should be noted that even the most complicated mathematical models in food web ecology are based on very simple ideas). Finally, although the historical context of progress in the field of food web ecology is central to this narrative, details are not elaborated in this text. The important idea is that at the present time, there are thousands of food web ecologists working on free-living species, but only very few parasitologists working on food webs. One can make a case that the field of food web ecology embodies the sum total of the methods, ideas, critical assumptions and theory that have been developed over nearly a century of scientific investigations by food web ecologists, *sans parasitologists*. Consequently, parasitologists seeking to work on food webs must necessarily learn the tools of the trade before they can contribute to the debate. The unfortunate by-product of this association is that many of the interesting questions from the parasitologists’ perspective tend to be overwhelmed and subsumed by the scientific issues and directions of the larger field. For example, when food web ecologists think about parasites in food webs, they are primarily interested in how parasites might affect the overall stability and persistence of the entire ecosystem as a functional unit. Almost all of their methods, analytical techniques and theories in the field are focused on elucidating these particular questions. On the other hand, when parasitologists think about food webs, they are primarily interested in the characteristics of the food web that allow successful colonization by parasites, and the ecological processes that contribute to the evolution of parasite life cycles and transmission pathways. This parasitological perspective is not as well-represented in the food web literature, but it will be the focus of this review.

Let us begin with the parasitological perspective on the title question, ‘where are the parasites in food webs?’ Food webs characteristically support many fewer parasite species than free-living species [8]. This might sound puzzling to some because the accepted dogma is that there are more parasite species than all other species on Earth combined. One of the first robust estimates of parasite diversity suggested that 70% of all animal species are parasites [15]. Even if one quibbles that this estimate was based only on British insects, subsequent studies using vastly different estimation parameters, confirm that there may be up to 50% more species enjoying a parasitic lifestyle than all other feeding strategies combined [16]. And even if one excluded the prions, bacteria, viruses, fungi, protozoa, and arthropods, there are still up to 300,000 helminth (worm) parasites in the world… and counting [17]. The best studied animal, *Homo sapiens,* serves as host to 342 different helminth parasites, and another 70 more if you count the protozoans [18].

So by all accounts, there are a lot of parasites out there in the world, yet it seems that food webs are only colonized by relatively few parasite species when compared to the numbers of available hosts. Some specific examples include the blackwater streams of New Jersey where the food web has 62 free-living species and 11 parasitic helminths [8,19,20]. At other locations in New Jersey, food webs in the Raritan River have 116 free living species and 21 parasites; and food webs in the Tuckerton salt marshes contain 92 free-living species and 16 parasites [21,22]. Some studies report greater proportions of parasites in food webs, e.g., in foodwebs from Carpinteria marsh (US) [23], tidal marshes in the Meadowlands (US) [24] or the Ythan estuary (Europe) [9]. The estimates are slightly higher in these food webs (up to 40% of total species) because, in addition to observed links (parasites that were actually recovered), these webs might also include putative links (parasites not actually recovered but thought to be there), and micropredators. In any case, regardless of the sampling or estimation methods employed in constructing food webs, or the type of food web ecosystem being studied, the numbers of parasite species are never greater than the numbers of free-living species in any food web. One might almost call this a general rule!

This pattern emerges because the same host species in different food webs may not always have the same parasites. More precisely, host populations are typically infected by only a small subset of their potential parasites. This is an explicit assumption of all parasitological models of epidemiology and transmission. As an extreme example, we do not expect to find all 342 helminth parasites of man in any one particular human population because human populations around the world each have their own distinct suites of parasites, depending on local geographical/ecological/economic conditions [18]. Nowhere is the idea that the same hosts in different food webs have different parasites more scrutinized than in fisheries management, an area where food web ecology and parasitology are most closely coupled. In this arena, it is well-established that accurate measures in fish migration patterns and stock assessment can be based on using parasites as biological tags [25-28]. Even fish stocks that are spatially or temporally separated by relatively short distances or times, can be identified by their parasites [29-31]. At the largest scale, anisakid nematode parasites of cetaceans are considered to have a world-wide distribution, but global parasite distribution maps indicate clearly that each anisakis species is restricted to specific areas within climate zones and oceans [32]. These data strongly suggest that food webs have structural or functional properties that facilitate the establishment of particular parasite species, depending on ecological circumstances. The nature of these structural/functional qualities of food webs that regulate parasitism is a principal question among parasitologists.

Parasitological models on the evolution of parasite life cycles often employ metaphors to illustrate concepts, and one such critical idea is the existence of ecological filters or barriers that a parasite must overcome before successfully parasitizing a new host [33,34]. The ‘encounter’ filter is related to the ecological circumstances that bring the incipient hosts and future parasites together in time and space. Since each parasite’s life cycle reflects evolutionary and co-evolutionary processes, this requires that all hosts in the parasite’s life cycle must occur in a stable configuration over very long periods of time for parasitism to evolve [34-37]. The whole community does not have to be stable, only those subgroups containing the appropriate hosts [34-36,38,39]. On the other hand, ecologists believed for a long time that there was an overall community configuration which promoted long-term stability and predictable dynamics that could be exploited by parasites [40]. The definition of stability also differs greatly between the two fields. Unlike ecological models where stability is an emergent property of complex networks, stability for parasitologists means that the hosts have to co-occur over a long time as “evolutionarily stable units” [36]. Typically, parasites are highly host-specific, and hosts are not interchangeable elements in parasite life cycles. Therefore, stable units that promote parasitism cannot be the result of cybernetic relationships that are randomly generated by the topological structure of the web.

The idea of stable sub groups within the overall community has also been considered in mainstream food web studies. A compartment, or module, is a theoretical construct that describes a subgroup containing resources, consumers, prey and predators, that behaves as an independent functional unit, and where the organisms are co-adapted to each other [41]. The debate on whether compartmentation existed in food webs has been going back and forth for several decades now [41-44]. The timing of this debate was unfortunate because the arguments were embedded in the larger quarrel over whether food webs were the result of highly interconnected species associating over long periods of time, or the result of random co-occurrences of organisms individually dispersing [44-46]. In the first view, the initial ecological circumstances and historical patterns of speciation and co-evolution are more important than the effects of local processes [47]. The second, and generally more theoretical view was that community assembly was largely a neutral process in which many species are ecologically equivalent, and the role of historical factors in community assembly was inconsequential [48]. This debate probably still continues in some parts, but recent investigations have silenced this dispute considerably, and the idea that communities are composed of interconnected subwebs has become an accepted paradigm [49-51]. Typical examples include plant/pollinator networks [52] and host/parasitoid and host/parasite networks [53,54]. A recent deconstruction of a large agrosystem identified 7 distinct subwebs, including a web between flowers and flower visitors; a web linking seeds, rodents and ectoparasites, and a web linking seeds, seed-feeding insects and parasitoids [55]. These authors were able to demonstrate mathematically that each subweb differed in their robustness or ecological stability, with some topological networks, e.g. those containing pollinators, that were particularly fragile.

### The food web matrix

Stability and subgroups are common themes in both fields, and so it seems natural that these two fields should easily come together, but this has not happened despite vigorous efforts from both sides [2,4]. The situation is probably not likely to get better because of the widely divergent directions of the two fields. In addition, there is a fundamental flaw in the food web methodology that severely limits the parasitological perspective. The essential problem is that the output of any analysis is only as good as the input, but the input data for most food web analyses are incomplete, and often inaccurate representations of nature. This criticism is chronicled in numerous previous publications [4,56], but I will summarize the two major points.

The primary analytical tool in food web studies is the food web matrix, where ‘topology’ refers to statistical patterns in the graph data of the matrix [57]. In a food web matrix, rows represent consumers (eg. predators), columns represent resources (eg. prey). The input data is binary. At the intersection of each row and column, a ‘1’ is assigned to the cell if the consumer feeds on the resource, and a ‘0’ is assigned to the cell if the consumer does not feed on the resource. To be completely clear about how the matrix is created, if a feeding connection exists between two species (eater and eatee), the link is given a value of 1, and if there is no feeding connection, the link is given a value of 0. The interesting thing is that in topological analyses, no additional measures of any other parameters are needed to calculate the key food web statistics or metrics! That is, with topology, there are no additional requirements for measures of interaction strength, or how often the species interact, or how many individuals of each species occur in the food web, or how many of one species are consumed by another, or the biomass and energy content of the participants in the community. The only data the food web matrix contain are the binary feeding connections, and nothing else [2,4,56,58]. As an extreme example, consider a food web where two predator species A and B, both feed on prey X. Both will be scored as 1 in the food web matrix even if the community consists of 5,000,000 individuals of species A and only 10 individuals of species B.

Of course, everyone realizes that it should matter how many individuals of each species inhabit the food web or the strength of the interactions between linked species, if we are to really understand the community. There have been numerous attempts to define or measure interaction strength between species, but there are none that can be universally agreed upon [59,60]. Even if we could agree on a good measure of interaction strength, this information would be very difficult to incorporate into the binary matrix. Much of the additional information on food webs has to be integrated *post hoc* on top of topological food webs to flesh out particular inferences. To make matters worse, there is a second big issue with the topological approach, the problem of taxonomic resolution. Incorrect taxonomy is the primary reason why the true number of players in food webs is rarely correct [61-64]. In theory, each species in the food web should have its own node, but the smaller, less charismatic, or difficult-to-identify species are often lumped together, resulting in a bias toward larger easier-to-identify species and higher trophic levels [2,4,63,65,66]. Poor taxonomy also means that the assignment of which-species eats-which-species in the matrix (0 or 1) can often be often unreliable or plain wrong [61], and feeding links are often based solely on body or gape size, or from reports in the literature rather than from observation [61,63,64,67]*.* The practice of lumping unidentified taxa together once sparked spirited debate in the field over whether the topology of the matrix could be affected by the resolution of food webs [61,68,69]. Of course, topological metrics will always be affected if nodes are added or removed from the matrix, and improving taxonomic resolution does significantly alter several key food web statistics, including connectance [63,68,70]. This should be a critical concern, but the debate has largely died down. In the end, it was not clear that increasing taxonomic resolution actually increased empirical rigor, and accurate taxonomy requires a huge increase in effort [68,71]. It seems clear that any analytical approach that calls for the identification of each individual species in the system as a distinct node, is operationally impossible. In addition, the identification of trophic links depends on extensive and detailed field observation and collection, complemented by laboratory rearing and feeding tests, but in a typical plant herbivore web containing 10,000 insect species and 200 host plants that are connected by 50,000 linkages, only 15% of the linkages meet the minimum criteria [64].

These criticisms of the topological approach may appear harsh, but they are the published opinions of the top practitioners in food web ecology [56,61,63,72-76]. Problems with the approach were brought into the spotlight by the recognition that food web patterns from real communities did not really support predictions from food web models [56,62,77,78]. Several noted that topology was the study of patterns in the graph data and statistics rather than the study of real patterns in nature [61,70,79,80], because the simple binary link approach does not accurately capture interactions in real food webs [61,81]. Focusing on food web statistics from topology may actually obscure real patterns in nature [82], and indeed, many spurious patterns in topology were hyped in the food web literature for prolonged periods, before being quietly discarded when their generality or accuracy was questioned [81,83,84]. For example, some of the most important patterns discovered in topological food webs were the scaling laws [85-87]. These supposedly constant ratios were believed to be insensitive to the size of food webs, but they were ultimately rejected as the resolution of the data improved, and the size of the database grew [56,81,84,88]. The problems with the topological approach are now openly acknowledged within the field, and even included in some of the latest textbooks [41,56]. Strangely, the dominance of topology persists in food web studies! The situation does not have an easy solution, but from the parasitological perspective, there is a serious concern that the focus on food webs through the narrow lens of topology will unnecessarily frustrate the understanding of parasites in food webs.

Why is it that we cannot give up on topological analyses? It is most likely because no one has invented a better way to analyze complex interaction networks. The most important parameters of ecological function in food web studies are stability, persistence and equilibrium, but these values can only be calculated from the topological matrix, i.e. in math terms, the community is stable if all the eigenvalues of the food web matrix have negative real parts [40]. This topological approach was adapted from graph theory in Physics, and it is proving difficult to conceptualize different or better analyses for complex systems [89]. Although everyone acknowledges the need to develop new approaches that incorporate measures of species abundance and interaction strengths [59,60], the use of topological analyses is being advocated as necessary to the iteration of the next generation of tools [62,90]. The truth is that no one really expects new analytical methods to materialize anytime soon because the true complexity of natural systems is overwhelming, and measuring interaction strength is challenging because of the large number of interactions, long-term feedback, and multiple pathways of direct and indirect effects that may potentially exist between species pairs [60,82]. However, perhaps the most important reason for the continued use of the topological approach is that it is endorsed by some of our most eminent theoretical ecologists [40,41,69]. Hence, for modern students of food web ecology, it remains acceptable to construct and think about food webs based only on linkage connectance.

How should parasitologists deal with this situation? A recent review by 10 of the top ecologists working on parasites in food webs referred to this as the “deep and central problem in theoretical biology and applied mathematics,” but still, they continue to advocate for the topological approach [4]. Most parasitologists are not sufficiently math-savvy to propose robust mathematical alternatives, and it can be easy to accept the *status quo*, especially since thousands of food web ecologists believe that topology is the most appropriate tool for deconstructing food webs. It is also hard to ignore topology because topological inferences are ubiquitous in the food web literature, and these studies are often buttressed by considerable ecological expertise and opinions in natural history that seem to validate the approach. In any case, whether realistic or not, the ideas generated from topological studies can sometimes be instructive, or at least thought-provoking to parasitologists. Thus, it would seem that the best way forward is to be extremely prudent in our endorsement of topological studies. Metaphorically-speaking, topology is like the skeleton of an animal. There can be a lot of useful information in skeletons, and the primary objective in food web theory appears to be mathematical/statistical algorithms that flesh out the animal. However, unlike dinosaur reconstructionists who are limited to fossilized skeletons, food web ecologists have the entire functioning animal, and they should use all of the data.

I will present new developments from both sides of this difference of opinion. Thus the following sections are organized under the headings of “Topological studies on parasites in food webs,” and “Non-topological studies and parasite life cycles.”

### Topological studies on parasites in food webs

The vast majority of papers in the field of food web ecology make use of topological analyses, and these studies are typically concerned with elucidating and predicting the big picture of ecosystem stability. For example, one can add or delete specific species in the matrix to determine the effects on overall system stability, and this is an important tool in fisheries management [91,92]. Since only binary data is required, the technique also makes it easier to quantify large scale ecological phenomena related to the effects of habitat destruction; species extinctions, alien invasions, and infectious disease epidemiology [93-95]. These areas of research on food webs are not discussed much in this review because most of these studies do not include parasites in their analyses, although this situation is changing as more parasitologists join the field [4,96]. Topological food web studies that do include parasites can be put into two general categories; studies that insert parasites into the matrix topology and in food web diagrams, and studies of parasites using network based analyses of webs and sub-webs.

### Parasites inserted into the matrix

When parasites were first inserted in food web matrix topologies, the most widely-reported finding was that they significantly altered several key food web metrics when compared to the same webs without parasites [4,9,10,19,23]. The list of altered food web metrics typically included increases in the number of links, increases in the linkage density (number of links per species), and increases in the values of connectance (the number of links/total links possible). Although these findings were often cited as solid evidence of the effects of parasites on food web functions [1,9,12,19], several authors have pointed out that this is essentially a non-result because the increases are an obvious result if you add 20-40% new species to the matrix [2,4,39]. These topology-based metrics are key parameters in the theoretical search for general patterns in food webs and as determinants of food web stability. Two of them, linkage density and connectance, are considered to be the most important statistics in food web topology because they are pivotal to system stability [23,41,56,97]. Adding parasites to the matrix significantly alters these two most important metrics, and this should mean something important about food web function… but no one seems to know how to interpret these changes [2,4]. For example, there is a strong theoretical link between connectance and system stability [98,99], yet the often-cited parasite-induced changes on connectance as they pertain to the stability of the system have yet to be explained [2,4,6,9,10,23,100].

When included in topological analyses, parasites are usually added as discrete nodes to the matrix, but this is not an entirely satisfactory solution for parasitologists. Parasites are not equivalent to predators (even though food web programs invariably place them as top predators in the analyses), and often have complex life cycle stages that infect different trophic levels. Sometimes, parasites are food themselves (accidental ingestion or transmission). One creative approach to deal with this analytical problem was the construction of parasite/host sub-matrices that can be analyzed independently of the main matrix [14,23]. However, the significance of the topological statistics derived from these sub matrices has not yet been validated, nor is it clear how the addition of separate parasite subwebs impinges on measures of overall system stability.

**Figure 1:**
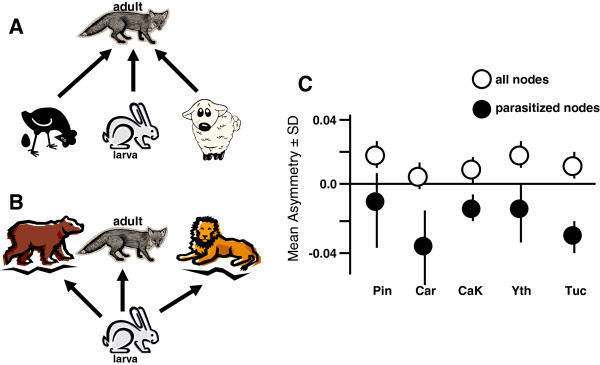
**Illustrations of A - negative asymmetry, and B – positive asymmetry at predator–prey nodes.** In these hypothetical examples, the perspective is that of a parasite whose larval stage is in the rabbit intermediate host, and adult stage in the fox definitive host. In this scenario, the other hosts are not parasitized. C. Summary of mean asymmetry values for all nodes (open circles) and parasitized nodes (closed circles) from 5 published webs; Pin- Pinelands NJ, Car – Carpenteria marsh CA; CaK- Carpenteria modified web CA; Yth -Ythan estuary, Scotland; Tuck –Tuckerton salt marsh, NJ by Rossiter and Sukhdeo 2011 [21].

There may be less traditional ways to estimate relative stability of host-parasite relationships from purely linkage data, at least from the perspective of parasites. Consider that linkages between predators and prey are rarely symmetrical. For example, at some nodes there may be only a few predator species feeding on many prey species (negative asymmetry; Figure 1A), while at other nodes, there may be a lot of predator species feeding on only a few prey species (positive asymmetry; typical of most predator prey nodes; Figure 1B). Parasites should preferentially exploit predator–prey interactions that are negatively asymmetric (e.g. few predator species feeding on many prey species) because these generalist predator hosts would be more stable over time, and would be less likely to have dramatic impacts on any one prey host, (including the intermediate host) [101,102]. At each node in the topological matrix, this asymmetry can be measured as the degree of ‘mismatch’ between the focal species and their associated interactions [103]. The nodal asymmetry of predator/prey interactions was investigated in five published food web topologies [21]. When looking at all predator/prey nodes in the web, the mean asymmetry values were positive in all of the 5 food webs (which means that on average, there were more predator species than prey species at each predator/prey node). However, if one considered only those nodes where parasite transmission occurred, the mean asymmetry values for those nodes were negative (more prey species than predator species at nodes using trophic transmission) in all of the five webs (Figure 1C).

It was interesting that parasites did not settle into predator–prey nodes that were symmetrically strong in both directions (only one predator and one prey at the node), although this guarantees a path where the intermediate host prey will always be eaten by the appropriate definitive host predator. Strong interactions between specialists often lead to “boom-bust” dynamics with high extinction risks for the species involved [104,105], and for parasites, these strong interdependent interactions may be less reliable over time. These data strongly suggest that parasitism is supported by specific structures in predator prey dynamics that may be related to stability of trophic transmission [21]. This is an exciting result, but the binary-biased analysis that we used in these studies should invite caution regarding our inferences. That is, the food web topologies in this study assume that all predator/prey links are of equal strength, and this is an unlikely scenario in nature.

### Parasites and network theory

The most recent but significant entrant into food web analyses is network theory, where topological features also characterize the structure and status of the network. The key parameters in network analyses reflect the social science origins of the technique [106-108], and include measurements of betweenness, closeness, nestedness, centrality, modularity, and node degree. The node degree (or connectivity; k) describes the number of links a single node makes with other nodes, and it is the most fundamental metric in these analyses. Like food web topology, network theory is also rooted in graph theory from Physics, and the data input is a binary matrix, so these analyses can be adapted to food web data.

#### Subweb networks

Subwebs typically only include a small subset of all species coexisting in a food web community, and network analyses of subwebs have yielded some interesting patterns. Network subwebs are called bi-partite interaction networks because they examine interactions between two guilds of interactors. They were first developed to study mutualists e.g., pollinators and flower species, where the number of visits by any species provides an estimate of interaction strength [109,110]. In most bi-partite networks there is a general pattern of high nestedness, or the degree to which species with few links have a sub-set of the links of other species, rather than a different set of links. This pattern emerges because most species in bi-partite subwebs turn out to be specialists. The few generalists have broad host ranges, and the networks are highly modular around highly interlinked core species [51,111-113]. With regard to parasites, there have been only a few analyses of host/parasite bi-partite networks, and these were based on matrices of linkage patterns between freshwater fish and their metazoan parasites, and between fleas and their mammalian hosts [53,54,114]. Host/parasite systems show the same patterns of nestedness and modularity seen in mutualistic plant/pollinator networks. That is, they are highly asymmetric, with specialist parasites tending to interact with hosts with high parasite richness, and hosts with low parasite richness tending to interact mainly with generalist parasites; resulting in high levels of nestedness and modularity [53,54,114]. Nestedness and modularity are generally thought to be the most important independent metrics in bi-partite networks, with potential implications on estimates of stability [54,109]. How these metrics may actually relate to stability is not very intuitive, and as the numbers of studies on bi-partite webs has grown (>75 and counting), this view is also being queried in some meta-analyses [115]. The real challenge now is how to assemble the component bi-partite networks into a whole food web network in a manner that is analytically tractable. In a well-resolved agrosystem containing seven distinct sub-webs with real interaction strength and energetic data, the complexity of the system still prevents easy integration of critical data into the topological analyses [55].

#### Whole web networks

**Figure 2:**
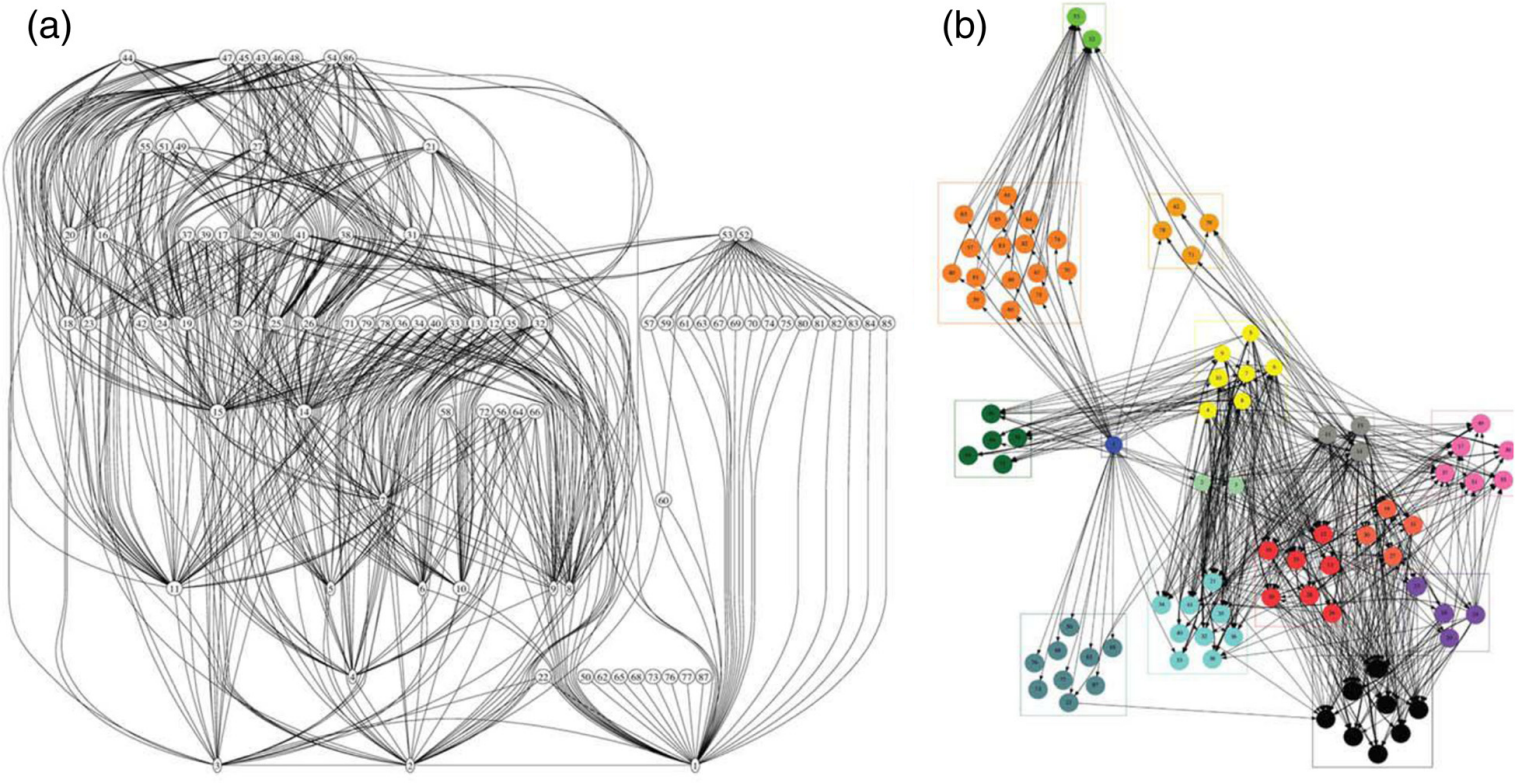
**Two analyses of the same topological data from a host food web in the Meadowlands salt marshes of New Jersey by Anderson and Sukhdeo 2011 [**[24]**].** (**a)** A traditional food web diagram showing linkages among participants. This is a parsimonious arrangement of species, so even though it seems as though there are 8 trophic levels, there are really only 4, with the graphing program spacing them out a little for the sake of visualization. (**b**) A network clustering algorithm partitioned the food web into 15 distinct modules of highly interacting species independent of trophic position, and suggested that parasites preferentially colonized highly connected modules of tightly interacting species which experience fewer fluctuations in abundance relative to those in the periphery.

Network analyses of whole food webs have also unearthed some potentially interesting patterns in topological structure [116-118], and certain features of the network may facilitate parasite colonization. For example, a recurrent pattern in food web networks is the presence of distinct cores, or hubs of highly linked species, which have been directly correlated with measures of system robustness [52,70]. With regards to parasites, most of the debate in the field has been centered on the question of how parasites might influence the structure and stability of food web networks [3,9,12,19,23]. However, several studies are now beginning to investigate how food web structures might affect the survival and persistence of parasites [97,100]. For example, in network analysis, grouping algorithms can be used to deconstruct complex organizational structures into clusters of interacting modules [119-121]. When applied to an exemplar host food web in the Meadowlands salt marshes of New Jersey, the algorithm partitioned the web into 15 distinct modules of highly interacting species, independent of trophic position (Figure 2b). Hosts in some modules were more heavily parasitized than in others, and the most consistent predictors were trophic generality and eigenvector centrality. This means that the parasites preferentially colonized host species that were highly connected, and which were contained within modules of tightly interacting species in the food web network [24]. These host species in the core module of a network may experience fewer fluctuations in abundance relative to those in the periphery, and this can provide a reliable host resource for parasites. These results, and the results of other network-based analyses that include parasites [53,54,114] suggest that highly connected free-living species interacting within core modules may represent stable trophic relationships that allow for the persistence of complex parasite life cycles [24]. These results also supports the notion that the topological structure of host food webs can have a significant effect on the establishment of parasites, and on the potential for evolution of complex parasite life cycles.

### Non-topological studies and parasite life cycles

**Figure 3:**
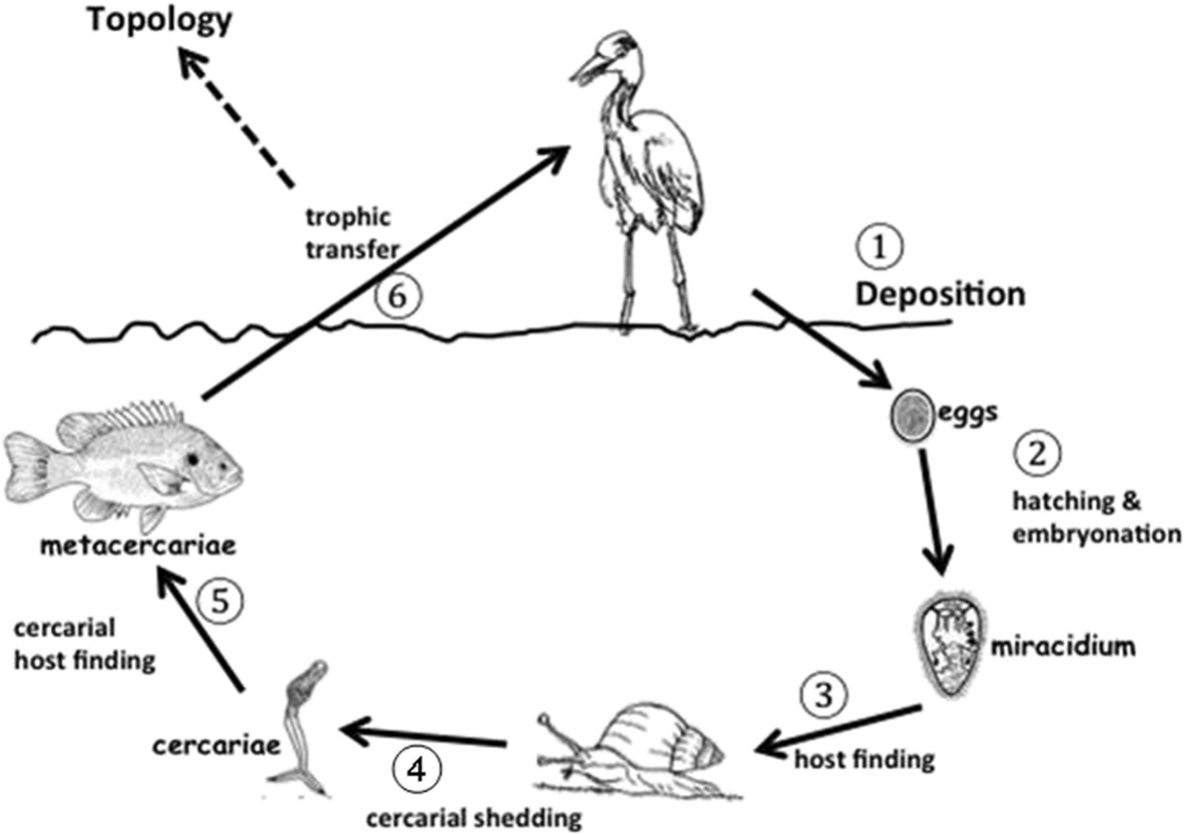
**The life-cycle of a typical trematode.** There are six distinct free-living parasite strategies during transmission from definitive host to definitive host that could only have evolved if there was an evolutionarily stable configuration between the hosts involved. Only one step, trophic transfer, is considered in the topological approach.

For parasitologists, there are other very compelling reasons why the topological approach and the modern food web perspective may be inappropriate (or more accurately, only partly appropriate) for the study of parasites. Consider the following example from one of the most successful groups of parasites on Earth, the trematodes. There are at least 6 distinct steps in the life cycle of trematodes where a stable community dynamic between the two hosts is required for the evolution of particular strategies during transmission (Figure 3). These include egg dispersal, embryonation and hatching, miracidial host-finding behavior, cercarial release, cercarial host finding, and trophic transmission. Trophic transmission is only 1 of these 6 important steps, but it is the only step considered in food web studies. Additionally, the vast majority of parasites on Earth have only one host [37], and direct life cycle parasites are also not considered in food web dynamics because they do not depend on trophic transmission. Clearly, restricting ourselves to studies of food web topologies that only measure “what eats what” might be the wrong way to think about parasites.

Let me use a specific example to drive this point home. In the host finding behavior of trematode cercariae (step 5 in Figure 3), the infective stage leaves the snail host and actively searches for the next intermediate host. However, there are usually NO feeding linkages between the 2^nd^ intermediate hosts (a fish in our example) and the 1^st^ intermediate hosts (snail) in trematode life cycles, so this particular step is ignored in food web topologies. Host finding behaviors in trematode cercariae are extremely specific to their hosts, and occur as hard-wired activities that bring these stages to the place where encounter with the next host is most probable [122-125]. By definition, the hardwired host-finding behaviors of cercariae are genetically fixed and neurobiologically invariant [122], and this behavioral ‘canalization’ requires prolonged periods where the presence of the hosts occurs in an evolutionarily stable configuration [122-125]. Food web theory has no explanation for why these two intermediate hosts should exist in a stable configuration because they are not topologically linked. Yet they clearly must somehow be linked in a stable and reliable manner, as evidenced by the hard-wired responses in the cercarial stages. One explanation for the strong relationship between these hosts might be strong indirect effects that are not measured in topologies, but which nevertheless leads to evolutionarily stable configurations that can be exploited by parasites. However, stable host configurations are required for the evolution of all 6 steps in trematode transmission strategies. For example, host-finding in the miracidial stages is also hard-wired and genetically fixed in the same way that cercarial behavior is fixed (step 3, Figure 3; [122,125]). From the perspective of miracidia, the upstream and downstream hosts are rarely linked in the food web sense (e.g., humans and snails in the life cycle of *Schistosoma mansoni)*, yet these two hosts must always occur in long term stable configurations for the genetically fixed behaviors in the miracidia to evolve. Food web theory also cannot explain this stable relationship between the definitive host and the 1^st^ intermediate host in trematode life cycles.

What are the available alternatives that might allow us to better explore parasites in food webs? In the topological world, the critical bottleneck is how to measure the interaction strength between two species, and how to appropriately include these measures into the matrix. The debate and controversy surrounding this issue is enormous [59,60] with several competing definitions and calculations for interaction strength. A sampling of these proposed metrics include measures of local species deletions [61,126,127], per capita interactions [59,67], Jacobian matrix elements [40,128], inverted matrices [129,130], energy flow [99,131,132], and Markov Chain models [133]. There are problems with most of these concepts because of potential indirect effects and unidentified non-linearities, and many are topology-based or situation-specific [59,60]. Parasites are rarely or never mentioned in these studies. I would argue that the best place to begin identifying realistic patterns in nature is to look at energy flow. Energy is a universal metric that can measure ecological costs and benefits for all stages in a parasite’s life cycle, and for every interaction (direct and indirect) that occurs in a food web.

**Figure 4:**
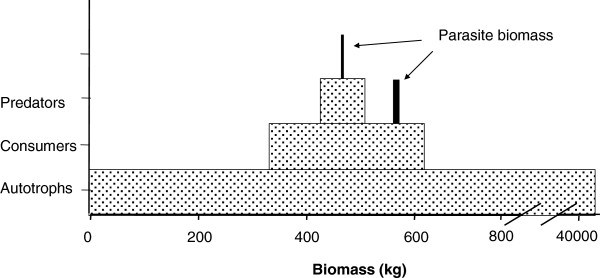
**Standing stock biomass patterns of autotrophs, consumers and predators in a food web recovered from a fairly pristine Pinelands stream by Hernandez and Sukhdeo 2008 [**[2]**,**[19]**]; this illustrates the ‘rule of ten’ in standing stock biomass pyramids.** Parasite biomass (in black) was recovered from two trophic levels in this system.

This ‘energetic perspective’ is really just a retelling of the old but still valid hypothesis developed 60–70 years ago by the fathers of food web ecology [134-137]. In fact, these ideas on energy are the foundation for all of modern food web theory [56,138-140]. In this view, energy is the currency of ecological interactions at all scales (from communities, to populations, to individuals), and its organizing principles are based on thermodynamic laws [99,135,138,141]. Indeed, the energetic perspective is the basis for the single uncontroversial law in food web ecology, which is often referred to as the “rule of ten” (Figure 4). That is, only ∼10% of the energy (typically measured as biomass) at any trophic level is transferred to the level above. This results in the classic pyramidal shape in trophic biomass that was first described by Lindeman, 1942 [134]. This pyramidal pattern in trophic biomass is a good place to start investigating energetics of parasites because this pattern is almost universal in food webs [99,141]. Rare exceptions to this pattern do occur e.g., biomass accumulation among plankton in highly productive marine systems can occur in a reverse pyramid because of differential growth rates [142].

### Parasites and the energetic perspective in food webs

The energetic perspective went out of fashion as theory gained dominance in the 70’s, but there have continued to be studies on diverse aspects of food web energetics, including direct estimates of energy flow and fluxes [143], stable isotope tracking [144], and stoichiometric patterns resulting from limitation of vital elements such as carbon, nitrogen, phosphorous or iron [145,146]. Most of these studies do not consider parasites. A major recent development is the metabolic theory of ecology (MTE), which proposes metabolism as a unifying principle for ecology in the same way that genetics underpins evolutionary biology [147]. Parasites seem to fit well within this theory [148], and the model’s metabolism-based allometric scaling laws may make it easier to estimate parasite energy fluxes in the web (Hechinger, pers. comm.).

At the most basic level, energy-oriented thinking about food webs can be traced back to the late 19^th^ century, and the essential idea is that all energy comes from the sun, is transformed into useable energy by plants and other autotrophs, and this energy percolates up the food web to every organism in the community. Only a very small fraction of the total biomass in the system is available to parasites (Figure 4), and in relation to host biomass, parasite biomass may account for only 0.2-1.3% of all animal biomass [20,149]. It might appear that parasite biomass fits nicely in the classic biomass pattern, but because parasites are not considered a distinct trophic level, they are not included under the rule of ten. Nevertheless, the important implication that emerges from these studies is that the flow of energy to parasites operates under the same thermodynamic rules that govern energy flows to every other organism in the food web [2]. Furthermore, since there is only a little energy available to parasitism, parasites probably engage in intense ecological competition for these limited resources. So, to partly answer the title question “where are the parasites in food webs?” the reason that parasites are not common in food webs (low parasite species richness) might be because the parasitic lifestyle is severely energy-limited. Indeed, in healthy ecosystems where energy flow is efficient and host diversity is high, more parasites are able to colonize the web [150-152]. A second answer to the question regarding where the parasites are, is that most of the parasite biomass in the web is localized within intermediate hosts (Figure 4). The standing stock of parasite biomass can be accurately estimated from measures of host population size together with prevalence and intensity data [153,154]. Intermediate hosts typically reside in the consumer (herbivore) trophic level, which represents a much larger proportion of overall system energy than definitive host predators, and this level can naturally support more parasite biomass.

**Figure 5:**
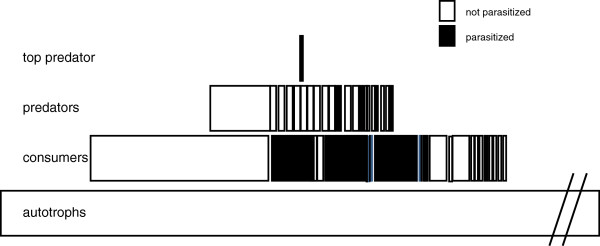
**Direct energetic measurements of net production (kj/m**^**2**^**/yr) values for each host species in a Pinelands stream food web based on bomb calorimetry.** Each compartment represents the total yearly production energy for each organism in the food web; the black compartments represent those hosts which are parasitized, Lettini and Sukhdeo, in prep.

**Figure 6:**
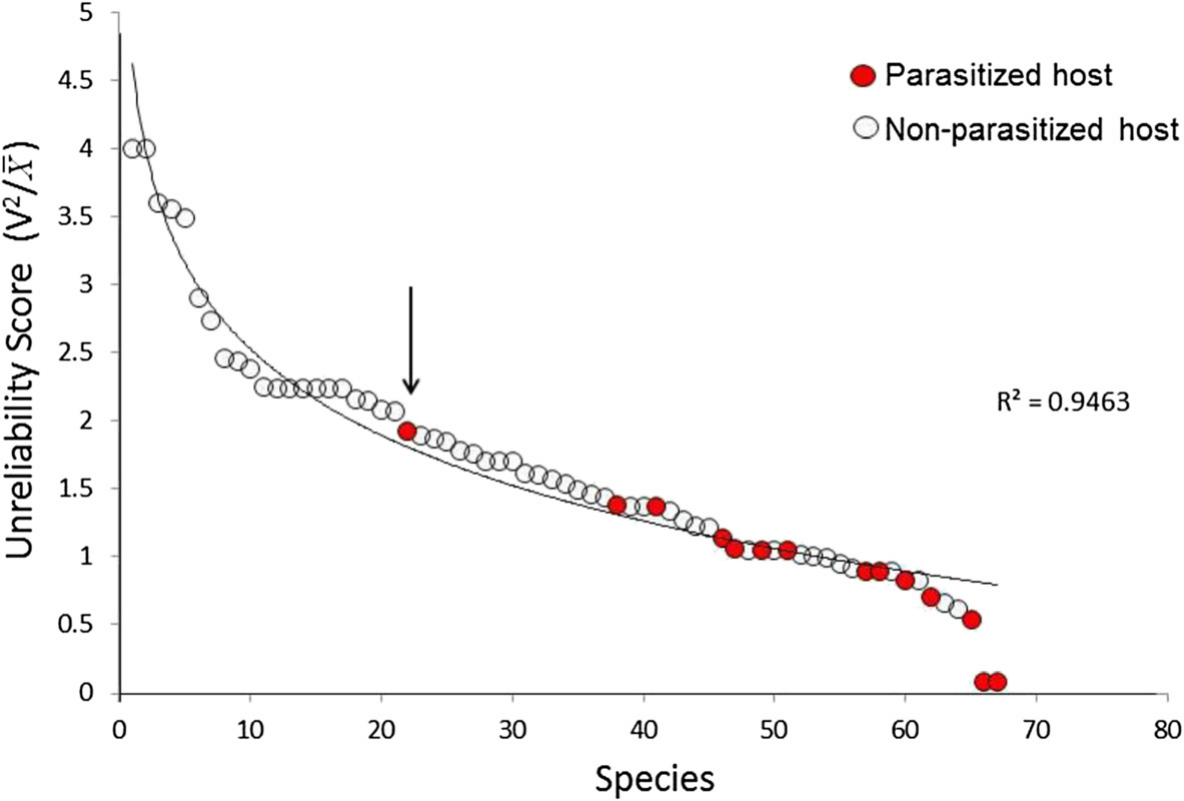
**Unreliability scores**$\left(V^{2}/\overset{―}{x}\right)$**for each of 68 host species in a Raritan river food web.** Total biomass for each species was measured for 8 consecutive seasons to determine biomass fluctuations used to calculate unreliability scores. Rossiter 2012 [22] found that parasitized hosts were among those hosts with the lowest unreliability scores. The parasitized host with the highest unreliability score (arrow) is a seasonal frog species.

Energetic patterns of biomass in the food web can inform parasite strategies. Take for example, the idea that only a few hosts in food webs are infected with parasites. One might imagine that the most energetically-abundant hosts might be the most favored by parasites, but this is not always so. Although parasites do seem to infect the most energy-containing hosts at any particular trophic level, they also infect hosts that do not appear to be sizeable energetic resources (Figure 5). One explanation might be that the total amount of energy in any potential host species is less important to parasites than the reliability or stability of the energy resource over time. The stability of hosts as energy resources can be estimated by the fluctuation or variation in host biomass over time, and biomass fluctuation was estimated in a food web containing 68 free-living organisms in the Raritan River in New Jersey. To arrive at a rigorous estimate of biomass stability over time, the variation in biomass of each species in the food web was measured for 8 contiguous seasons over two years. A simple “Unreliability” index $\left(V^{2}/\bar{x}\right)$ was used to measure relative variability in biomass; high values of the index mean that there is a lot of variation in individual biomass and thus the host is unreliable, low values mean greater stability in host biomass over the two years. The data show that parasites preferred hosts that were among the most stable in their seasonal biomass values (Figure 6), clearly supporting the idea that reliable and stable energetic resources are an important prerequisite for parasitism.

**Figure 7:**
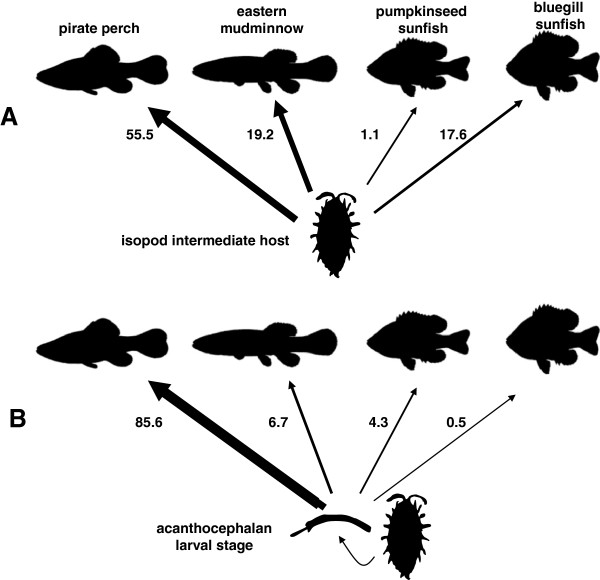
**A. Hernandez and Sukhdeo 2008 [**[19]**] measured the proportion (%) of isopod intermediate hosts in the diets of four different hosts as determined from stomach content analysis. B.** Prevalence of infection (%) with the acanthocephalan parasite *Acanthocephalus tahlequahensis* whose larval stage is found in the isopod *Ceacidotea communis*. Bluegill sunfish are recent invaders in this system and are relatively resistant to the parasite; this is a good example of the dilution effect where adding non-competent hosts to the system compromises the transmission rate of the parasite to its normal host.

Another simple method of evaluating energetic stability or reliability in host resources is to track parasites as they follow the flow of energy in the web. Intermediate host prey often have several predators, each of which can serve as definitive hosts. Given a choice, parasites should choose hosts with the most reliable flows of energy. Energetic pathways and energy flows in food webs are determined by what each organism eats. There are many techniques available to measure intake, from simple analyses of stomach contents to sophisticated techniques such as stable isotope analysis or fatty acid signatures to measure what animals eat [155]. These methods have been used to track the sealworm *Pseudoterranova decipiens* up the food chain [156], to determine the effects of intermediate host species loss on parasite richness [157], and to identify the feeding interactions in fish via their parasites [38,158-160]. A good example is the marine grenadier fish, which changes its diet as it gets older and larger, and the parasite species that infect each of these age classes are directly correlated to the consumption of specific intermediate host species [161]. A similar analysis on an acanthocephalan parasite of freshwater fish showed that adult parasite prevalence in four potential definitive hosts is proportional to the consumption of the intermediate isopod (Figure 7). In this study, pirate perch is the native fish species and it is the normal definitive host of the parasite. The pirate perch is an efficient predator on this particular isopod species, *Ceacidotea communis*, and the flow of energy from intermediate host to potential definitive hosts in the food web clearly have an impact on the parasite’s life cycle strategy.

It is clear that there are many ways in which an energetic perspective can illuminate parasite biology from the individual to the community levels. For example, egg production is a high energy activity and one of the first steps in parasite transmission, yet it is rarely considered in the energetics of food webs. Parasite numbers can be very impressive considering how small some of these worms are. The daily egg production in gastrointestinal nematodes of domestic animals might be as high as 10,000 eggs/day in *Oesophagostomum* or *Chabertia* spp., the trematode *Fasciolopsis buski* lays 25,000 eggs/day, and females of the incredible *Ascaris lumbricoides* produce 230,000 eggs/day, for a total of 27,000,000 eggs produced in a single female’s lifetime [162]. Where does the energy come from? Where does it go? These questions can be applied for each step in the life cycle. However, is not clear how, or if at all, these consistent energetic drains affect food web function, and more importantly, if these energetic costs affect the parasites’ choice of hosts. There are also significant indirect energetic costs caused by altered energy allocation to maintenance, reproduction, and respiration in the host [163-166]. Infected hosts tend to increase their metabolic rate, deplete their energy reserves and increase their ecological efficiency [160,164]. These costs are seemingly tiny in the overall ecosystem energy budget, but they may be critical in the success or failure of particular parasite strategies.

At the level of individual hosts, we can precisely measure the energetics of individual parasites using sophisticated devices, and several studies confirm that only small quantities of energy are extracted from individual hosts [20,167-169]. For example, bomb calorimetry of the isopod *Ceacidotea communis* parasitized by the larvae of *Acanthocephalus tehlequahensis* show that individual infected isopods allocate as much as 20% of their production energy to parasite growth [20]. The costs on these individual hosts can be scaled up to the population level (infected and uninfected) based on parasite prevalence to show that at least 7.0% of the production energy of the entire isopod population in the stream is diverted towards this parasite [20]. These negative effects of parasites on individual hosts can trickle up to even higher ecological levels and can have significant impacts upon the entire community [170,171]. For example, isopods infected with the acanthocephalan parasite significantly reduce their detritus-processing, and this significantly reduces the availability of nutrients to all other organisms in the entire stream ecosystem [172].

### Energetics is not enough!

Although the energetic perspective provides a historically and intellectually solid theoretical paradigm for generating testable hypotheses on parasites, by itself, it is not sufficient to explain the full roles of parasites in food webs. For example, energetics cannot answer questions on why most hosts in trematode life cycles are not linked energetically, yet they form long term stable configurations that are the bases for the evolution of hard-wired behaviors in both miracidia and cercaria stages. There are also several critical indirect costs that cannot be measured using energetic parameters, but which may be extremely significant to the hosts. For example, as is often the case in acanthocephalan life cycles, the larval acanthocephalan parasite described above castrates its isopod host. Here, the direct energetic changes are easy to measure (infected isopods allocate zero production energy to reproduction), but the biological costs of the lost reproductive capacity due to parasitic castration are almost impossible to quantify in terms of energy [20].

**Figure 8:**
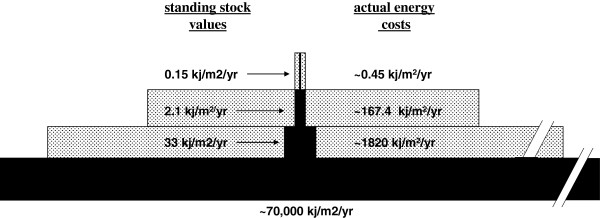
**Typical pattern of standing stock biomass pyramids of a black water stream in New Jersey (black), and the estimates of actual production energy at each trophic level (grey).** Parasites were included in the appropriate predator and consumer trophic levels. These data suggest that the real energetic costs at lower trophic levels (grey) can be significantly higher than estimates according to the rule of 10 (Lettini and Sukhdeo, in prep.).

There is a need for new and different ways of thinking, and this may require that parasitologists have to challenge hard-core beliefs. It is often difficult to change inaccurate or flawed ideas that represent long-held truths, but all ideas should be carefully scrutinized, including even ecology’s most famous rule, the rule of ten. There is an important challenge to this rule by marine and freshwater biologists working to develop accurate estimates of biomass conversion [173]. In the rule of ten, visualized as the classic biomass pyramid, the pattern is constructed from measurements of standing stock biomass (Figure 4). Hydrobiologists contend that this method does not provide an accurate picture of system energetics because it does not take into account the differential rates of biomass turnover for different organisms. A good metaphor for this is that standing stock biomass is like the balance in a checking account; the balance can remain steady at $100 even though thousands of dollars may have been deposited (production energy) and spent during the year. The total amount deposited (production energy) is the balance in the account (standing stock) multiplied by the number of times the standing stock has been spent and replenished, i.e. the turnover rate. Thus, the actual costs involved in trophic production must take into account the turnover rate of organisms, and small animals at the bottom of the food web turnover much more quickly than large animals at the top of the food chain. Turnover rates, often defined as (P/B); annual production (P) divided by mean biomass (B) can vary from <1 for top predators to >100 for some macroinvertebrates [174]. For example, P/B ratios for midge larvae in streams can be as high as 200, and thus, biomass standing stock values underestimate the production energy in this organism 200-fold [175]. Another way to think of this is as the resident time of energy at each trophic level, or the time it takes energy to flow through the ecosystem [176]. Average resident times in marine ecosystems range from about “6 days for phytoplankton to 2 months for zooplankton, to 4 months for cephalopods, 8 months for crabs and shrimp, 1.5 years for fish, 15 years for seals and 50 years for whales” [177]. This new interpretation will fundamentally changes our ideas on the energetic costs of life, and it will have significant repercussions on all previous estimates of energy flow in food webs. We applied these analyses to a stream food web to provide a visual example of relationship between standing stock measurements in traditional food web studies versus the actual production energy costs involved (Figure 8). The pattern of the real energetic pyramid suggests that while the rule of 10 may apply to trophic transfer at the top of the food web where energy transfer is more efficient, but not at the lower trophic levels where the production energy required to sustain trophic biomass is considerably higher than is generally considered [171]. It seems clear that production energy more accurately reflects the true energetic costs of trophic transfer, and there is mounting pressure to use production energy rather than its surrogates, density or biomass, in studies of energy flow in food webs [171]. This is a huge challenge to food web theory.

## Conclusions

In conclusion, this review posits that traditional food web approaches based on topological analyses do not take into account all aspects of parasite life cycles. Whereas the energetic approach provides an alternate platform to evaluate the role of parasites in food webs, neither of these approaches is sufficient by themselves. For parasitologists, there is a clear need for creative methods to decipher the ecological processes that contribute to the evolution of parasite life cycles and transmission pathways, and it seems obvious that new insights will come from empirical investigations of real food webs rather than from mathematical theory. Ecology is founded on the search for, and explanation of, patterns in nature [178], and elucidating new patterns will require careful observation and experimentation within the perspective of natural history. Parasitologists are in position to lead the way simply because their parasite identification skills are a limited resource, and parasitologists think about food webs from the point of view of the parasite rather than from the point of view of the host. This parasite-centric perspective, or the worm’s eye view, has already identified new patterns in food web energetics that challenge conventional wisdom and illuminate our understanding of parasite biology.

## Competing interests

The author declares that he has no competing interests.

## Authors’ contributions

Sole author.
